# ZD6474 reverses multidrug resistance by directly inhibiting the function of P-glycoprotein

**DOI:** 10.1038/sj.bjc.6603985

**Published:** 2007-10-02

**Authors:** Y Mi, L Lou

**Affiliations:** 1 Shanghai Institute of Materia Medica, Chinese Academy of Sciences, Shanghai 201203, China

**Keywords:** ZD6474, P-glycoprotein, multidrug resistance

## Abstract

P-glycoprotein (P-gp) pumps multiple types of drugs out of the cell, using energy generated from ATP, and confers multidrug resistance (MDR) on cancer cells. ZD6474 is an orally active, selective inhibitor of the vascular endothelial growth factor receptor, epidermal growth factor receptor, and rearranged during transfection tyrosine kinases. This study was designed to examine whether ZD6474 reverses P-gp-mediated MDR in cancer cells. Here, we show that clinically achievable levels of ZD6474 reverse P-gp-mediated MDR of the P-gp-overexpressing cell lines derived from breast cancer, MCF-7/adriamycin (ADR), and human oral epidermoid carcinoma, KBV200 to ADR, docetaxel, and vinorelbine. This ability to reverse the P-gp-mediated resistance is comparable to that of another frequently used reversal agent known as verapamil. ZD6474 itself moderately inhibits the proliferation of both MCF-7 and MCF-7/ADR cells with almost equal activity, but its inhibitory effect is not altered by co-incubation with verapamil, suggesting that ZD6474 may not be a substrate of P-gp. In addition, ZD6474 increases the intracellular accumulation of the P-gp substrate, rhodamine-123, and ADR, by enhancing the uptake and/or decreasing the efflux of these compounds in resistant cells. Further studies show that ZD6474 stimulates ATPase activity in a dose-dependent manner, which is required for the proper function of P-gp. In contrast, ZD6474 does not inhibit the expression level of P-gp. Our results suggest that ZD6474 is capable of reversing MDR in cancer cells by directly inhibiting the function of P-gp, a finding that may have clinical implications for ZD6474.

Transporter proteins belonging to the ATP-binding cassette (ABC) superfamily pump out drugs using the energy from ATP hydrolysis and this leads to resistance of cancer cells to multiple anticancer drugs (Bates *et al*, 2001; Borst and Elferink, 2002). ABC transporters are frequently expressed in tumour tissues, so it is believed that the overexpression of ABC transporter proteins is a major mechanism responsible for multidrug resistance (MDR) (Litman *et al*, 2001). Among them, P-glycoprotein (P-gp/ABCB1) plays a key role in mediating MDR (Borst and Elferink, 2002; Doyle and Ross, 2003).

ZD6474 (Vandetanib; ZACTIMA, AstraZeneca Pharmaceuticals, Macclesfield, UK) is an oral, small molecule inhibitor of the vascular endothelial growth factor receptor (VEGFR), epidermal growth factor receptor (EGFR), and rearranged during transfection (RET) tyrosine kinases. ZD6474 displays antitumour efficacy by directly inhibiting tumour cell proliferation and survival *via* EGFR and RET inhibition, as well as tumour angiogenesis *via* VEGFR inhibition (Ryan and Wedge, 2005). ZD6474 is currently in phase III clinical trials for the treatment of follicular, medullary, anaplastic, and locally advanced and metastatic papillary thyroid cancer, as well as for other cancers including non-small cell lung cancer.

Multidrug resistance is one of the major causes of failure in cancer chemotherapy, and numerous efforts have been made to overcome MDR. The most employed strategy has been to develop MDR inhibitors including the calcium channel blocker, verapamil (VPL), and the immunosuppressant, cyclosporine A, which reverse MDR by functioning as competitive substrates of P-gp. However, their clinical benefits are limited due to high toxicity at resistance-inhibiting doses. Another important MDR inhibitor, PSC-833, has not been successful in clinical trials either. Recent studies have shown that the tyrosine kinase inhibitors, STI-571 and AG1393, interact with the human P-gp and human multidrug resistance protein 1 (ABCC1) (Hegedus *et al*, 2002), and significantly inhibit ABC transport activities. Gefitinib, an EGFR inhibitor, reverses chemotherapy resistance in multidrug-resistant cancer cells expressing the ABC family of proteins (Yang *et al*, 2005). These reports collectively suggest that tyrosine kinase inhibitors may be promising MDR inhibitors. More importantly, it has been reported that ZD6474 has synergistic effects with many chemotherapeutic agents in reducing colony formation in GEO, MCF-10A ras, ZR-75-1, OVCAR-3, CALU-6, MNK-28, and AGS cancer cell lines grown in soft agar (Ciardiello *et al*, 2003) *in vitro*. *In vivo*, ZD6474 may potentiate the efficacy of paclitaxel against established human gastric carcinoma GEO xenografts (Ciardiello *et al*, 2003). Currently, ZD6474 is being clinically tested for its efficacy in combination with multiple chemotherapeutic agents (Massarelli and Herbst, 2006). Nevertheless, the mechanism underlying the ZD6474-mediated chemosensitising effect is still unclear. In this study, we examined the effect of ZD6474 on MDR and found that ZD6474 reverses the P-gp-mediated resistance to adriamycin (ADR), docetaxel, and vinorelbine (VNR), but increases the accumulation of P-gp substrates in multidrug-resistant MCF-7/ADR cells. This is achieved by stimulating the ATPase activity of P-gp that is expressed on the cell membrane. Our findings suggest that a combination of ZD6474 with cytotoxic agents may overcome MDR in clinical settings.

## MATERIALS AND METHODS

### Materials

*Chemicals and reagents* ZD6474 was synthesised at the Shanghai Institute of Materia Medica, Chinese Academy of Sciences, China. Docetaxel, ADR, and VNR were obtained from Jiangsu Hengrui Pharmaceutical Co. (Lianyungang, China). Sulforhodamine B, rhodamine-123 (Rho-123), and VPL were purchased from Sigma Chemical Co. (St Louis, MO, USA). The antibody to P-gp was purchased from Alexis Biotechnology Inc. (San Diego, CA, USA).

*Cell culture* The human breast cancer cells, MCF-7, and their MDR cell subline MCF-7/ADR were maintained in Dulbecco's modified Eagle medium containing 10% heat-inactivated fetal bovine serum (FBS), 100 kU l^−1^ penicillin, 200 kU l^−1^ streptomycin, and 0.01 mg ml^−1^ bovine insulin at 37°C (5% CO_2_). The human oral epidermoid carcinoma cell line, KB, and its MDR cell subline, KBV200, were maintained in RPMI 1640 medium containing 10% heat-inactivatedFBS, 100 kU l^−1^ penicillin, and 200 kU l^−1^ streptomycin.

*Cytotoxicity test* Cytotoxicity tests were performed using a sulforhodamine B assay (Schneider *et al*, 1994). Briefly, cells were grown in 96-well plates and various concentrations of drugs were added to the wells for 72 h prior to being assayed. The IC_50_ (concentration required for 50% inhibition) was calculated by the Logit method. Fold resistance for individual drugs was defined as the IC_50_ of MDR cancer cells divided by that of the parental drug-sensitive cells.

*Drug uptake and efflux assay* The assay was performed according to a previously described method (Ludescher *et al*, 1991; Wang *et al*, 2004; Yang *et al*, 2005). For the Rho-123 uptake assay, cells were incubated with 5 *μ*M Rho-123, in the presence or absence of ZD6474, at 37°C for 1 h. The cells were observed and photographed under an Olympus fluorescence microscope (Olympus Optical, Tokyo, Japan) or analysed with a FACSCalibur flow cytometer (BD Biosciences, San Jose, CA, USA). For the efflux assay, the cells were incubated in medium containing 1 *μ*M ADR for 1 h, and then cultured in ADR-free medium, with or without ZD6474 for additional 1.5 h. Cellular ADR accumulation was then analysed as described above.

*Preparation of cell lysates and western blotting* After drug treatment, cells were washed twice with ice-cold phosphate-buffered saline (137 mM NaCl, 2.7 mM KCl, 10 mM Na_2_HPO_4_, 1.8 mM KH_2_PO_4_, pH 7.4) and total cell lysates were collected in sodium dodecyl sulfate (SDS) sample buffer (50 mM Tris-HCl, pH 6.8, 100 mM dithiothreitol (DTT), 2% SDS, 0.1% bromophenol blue, 10% glycerol). Cell lysates, containing equal amounts of protein, were separated by SDS–polyacrylamide gel electrophoresis (PAGE) and transferred to polyvinylidine difluoride membranes. After being blocked in 5% non-fat milk in Tris-buffered saline with 0.1% Tween 20 (pH 7.6), membranes were incubated with the appropriate primary antibodies at 4°C, overnight, and exposed to the appropriate secondary antibody for 3 h at room temperature. Immunoreactive proteins were visualised using the enhanced chemiluminescence system from Pierce (Rockford, IL, USA).

*Measurement of P-gp ATPase activity* MCF-7 and MCF-7/ADR cells were harvested, and their membranes were isolated and stored at −80°C as described previously (Orlowski *et al*, 1996; Garrigues *et al*, 2002). ATPase activity was measured at 37°C, with a coupled enzyme assay, by continuous monitoring of absorbance at 340 nm. In order to maintain constant MgATP concentrations, the medium consisted of 10 mM Tris/HCl, pH 7.8 (at 20°C), 100 mM NaCl, 10 mM KCl, 2 mM MgCl_2_, 1 mM DTT, and 1 mM MgATP, and was supplemented with 1 mM phosphoenolpyruvate, 0.5 mM nicotinamide adenine dinucleotide, 0.1 mg ml^−1^ lactate dehydrogenase, and 0.1 mg ml^−1^ pyruvate kinase. Some membrane ATPases were inhibited by the addition of 10 mM sodium azide, 0.5 mM ouabain, and 1 mM ethylene glycol tetraacetic acid.

### Statistical analysis

The paired Student's *t*-test was used for significance where indicated.

## RESULTS

### ZD6474 reverses MDR

**Figure 1:**
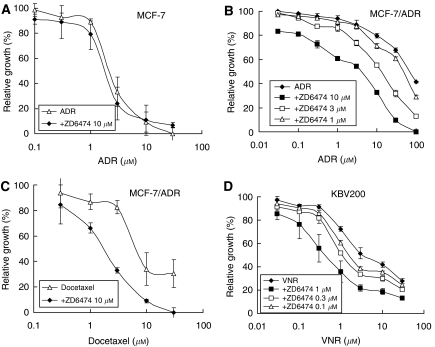
ZD6474 reverses P-gp-mediated resistance. P-gp-negative MCF-7 cells or P-gp-positive MCF-7/adriamycin (ADR) and KBV200 cells were treated with (ADR, docetaxel, or vinorelbine (VNR) in the presence or absence of ZD6474 for different periods of time, and the cell proliferation was determined by the SRB assay. Each point represents the mean±s.d. for three determinations.

P-glycoprotein is overexpressed in MCF-7/ADR and KBV200 cells (data not shown). The IC_50_s of ADR for MCF-7 and MCF-7/ADR cells were 2.0 and 122.8 *μ*M, respectively (Figure 1A and B). Thus, overexpression of P-gp confers MCF-7/ADR cells resistance to ADR (>60-fold resistance). In addition, MCF-7/ADR cells are simultaneously resistant to docetaxel (data not shown), demonstrating that MCF-7/ADR cells have an MDR phenotype. ZD6474 did not sensitise MCF-7 cells to ADR (Figure 1A), but significantly sensitised MCF-7/ADR cells to ADR and docetaxel, as well as sensitising KBV200 cells to VNR (Figure 1B–D). The chemosensitising ability of ZD6474 was dose dependent (Figure 1B and D). The IC_50_s were dramatically decreased by 10 *μ*M ZD6474, from 122.8 to 4.1 *μ*M for ADR, and from 9.4 to 1.7 *μ*M for docetaxel (Figure 1B and C). ZD6474 (1 *μ*M) also caused a decrease in the IC_50_ of VNR, from 5.7 to 0.6 *μ*M (Figure 1D) in KBV200 cells. Of note, 10 or 1 *μ*M ZD6474 itself had little growth-inhibitory effect on MCF-7/ADR cells or KBV200 cells, respectively. These results suggest that ZD6474 is capable of reversing P-pg-mediated MDR and that this effect is probably mediated *via* P-gp inhibition.

### ZD6474 may not be a substrate of P-gp

**Figure 2:**
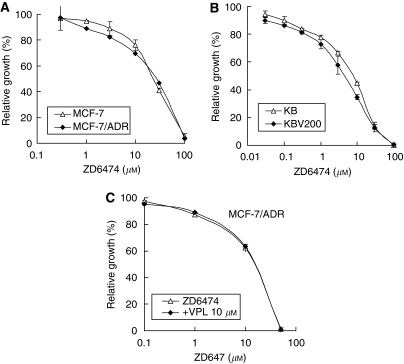
ZD6474 may not be a substrate of P-gp. (**A**, **B**) P-gp-negative MCF-7 and KB cells or P-gp-positive MCF-7/adriamycin (ADR) and KBV200 cells were exposed to ZD6474 for 72 h. (**C**) MCF-7/ADR cells were treated with ZD6474 in combination with verapamil for 72 h. The cell proliferation was determined by the SRB assay. Each point represents the mean±s.d. for three determinations.

ZD6474 itself has an equally moderate inhibitory effect on the proliferation of both MCF-7 and MCF-7/ADR cells. As shown in Figure 2A, ZD6474 inhibited the proliferation of MCF-7 and MCF-7/ADR cells in a dose-dependent manner, with IC_50_s of 17.3 and 19.3 *μ*M, respectively. This was also the case in KB and KBV200 cells where the IC_50_s were 5.4 and 4.4 *μ*M, respectively (Figure 2B). These data indicate that overexpression of P-gp does not confer cell resistance to ZD6474, and ZD6474 may not be a substrate of P-gp. To confirm this, we determined the effect of VPL on ZD6474 in P-gp-overexpressing MCF-7/ADR cells. VPL, a substrate of P-gp, did not influence the inhibitory effect of ZD6474 in MCF-7/ADR cells (Figure 2C). These data collectively demonstrate that ZD6474 may not be a substrate of P-gp.

### ZD6474 modulates P-gp-mediated uptake and efflux

**Figure 3:**
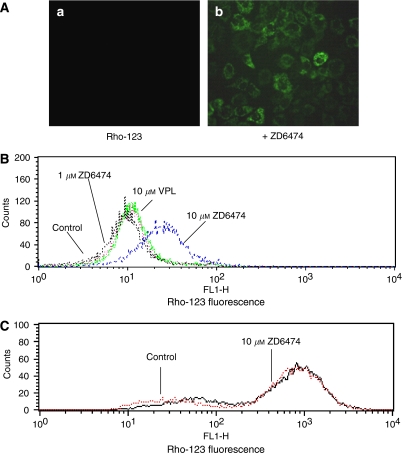
Effect of ZD6474 on the uptake of rhodamine-123 (Rho-123) in MCF-7/ADR cells. (**A**) MCF-7/ADR cells were incubated with 5 *μ*M rhodamine-123 in the absence (a) or presence of 10 *μ*M ZD6474 (b) at 37°C for 1 h. The cells were observed and photographed under a fluorescence microscope. (**B**) Uptake of rhodamine-123 in multidrug-resistant MCF-7/ADR cells was analysed by flow cytometry. (**C**) ZD6474 had no evident effect on the rhodamine-123 uptake in MCF-7 cells that do not overexpress P-gp. Data shown are representatives of at least three independent experiments.

**Figure 4:**
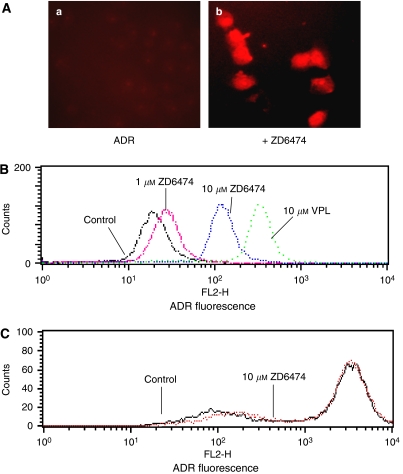
Effect of ZD6474 on the efflux of adriamycin (ADR) in MCF-7/ADR cells. (**A**) MCF-7/ADR cells were incubated with 1 *μ*M ADR at 37°C for 1 h, then incubated in ADR-free medium without (a) or with 10 *μ*M ZD6474 (b) at 37°C for an additional 1.5 h to allow efflux of ADR. The cells were observed and photographed under a fluorescence microscope. (**B**) Efflux of ADR in multidrug-resistant MCF-7/ADR cells was analysed by flow cytometry. (**C**) ZD6474 had no evident effect on the ADR efflux in MCF-7 cells that do not overexpress P-gp. Data shown are representatives of at least three independent experiments.

Reversal of MDR is usually manifested as an increased intracellular accumulation of chemotherapeutics, which can be achieved by disturbing P-gp-mediated drug uptake and efflux. Therefore, we examined the effect of ZD6474 on the uptake and efflux of two substrates of P-gp, Rho-123, and ADR, in MCF-7/ADR cells. ZD6474 was shown to increase Rho-123 uptake and decrease ADR efflux in MCF-7/ADR cells in a dose-dependent manner as determined by image analysis (Figure 3A and 4A) and this was confirmed by flow cytometry analysis (Figure 3B and 4B). The fluorescent index of Rho-123 was increased from 12.0 to 28.7 by 10 *μ*M ZD6474 (Figure 3B). Similarly, the fluorescent index of ADR was increased from 21.7 to 107.6 by 10 *μ*M ZD6474, and to 286.5 by 10 *μ*M VPL (Figure 4B). In control MCF-7 cells, which do not overexpress P-gp, ZD6474 had no evident effect on uptake of Rho-123 nor efflux of ADR. (Figure 3C and 4C).

### ZD6474 stimulates P-gp ATPase activity, but does not affect P-gp expression

**Figure 5:**
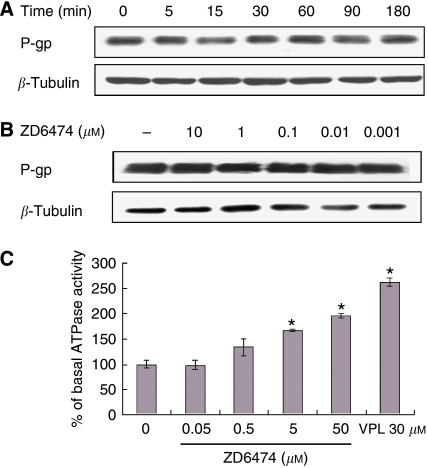
ZD6474 stimulates P-gp ATPase activity, but does not affect P-gp expression. (**A**) MCF-7/ADR cells were exposed to ZD6474 for 3 h. (**B**) MCF7/ADR cells were treated with ZD6474 for different periods of time. Cell lysates were resolved by SDS–PAGE and immunoblotted with antibodies specific for P-gp. Data shown are representatives of at least three independent experiments. (**C**) P-gp-expressing membranes were obtained from MCF-7/ADR cells and the ATPase activity was determined as described in the ‘Materials and methods’ section. Each point represents the mean±s.d. for three experiments. ^*^P<0.05 *vs* control by Student's *t*-test.

The increased accumulation of intracellular drug may be a result of decreased expression of P-gp or increased activity of P-gp. Therefore, we examined the effect of ZD6474 on both the expression and activity of P-gp. As shown in Figure 5A and B, the expression level of P-gp was not affected by different treatments with ZD6474. However, ZD6474 significantly increased the ATPase activity, in a dose-dependent manner, in membranes of P-gp-overexpressing MCF-7/ADR cells (Figure 5C), but had no detectable effect on the ATPase activity in MCF-7 membranes, which do not express P-gp (data not shown).

## DISCUSSION

ZD6474 is an oral, small molecule inhibitor of VEGFR, EGFR, and RET tyrosine kinases. Unlike other chemotherapeutic agents that readily develop cross-resistance towards other structurally related or unrelated compounds, both *in vitro* and in the clinic, ZD6474 has not been reported to develop cross-resistance to prior chemotherapeutic agents, either *in vivo* or *in vitro* (Morelli *et al*, 2005). In good agreement with these reports, the present study shows that ZD6474 displays almost equal activity in P-gp-negative and -positive breast carcinoma and oral epidermoid carcinoma cells (Figure 2). These results indicate that P-gp does not confer resistance to ZD6474 in cancer cells and that ZD6474 has no cross-resistance to classical cytotoxic agents such as ADR, docetaxel, and venorelbine. This is of clinical significance because it suggests that ZD6474 may be a good option for patients already developing MDR.

More importantly, we not only found that ZD6474 may not be a substrate of P-gp, but that it is also a good inhibitor of MDR. This is based on the following facts. ZD6474 only increases intracellular accumulation of Rho-123 and ADR, which are substrates of P-gp, and sensitises chemotherapeutic agents in cells such as MCF-7/ADR and KBV200 both of which overexpress P-gp, but not in cells such as MCF-7 and KB both of which do not express P-gp. More importantly, ZD6474 stimulates the ATPase activity of P-gp, which is a key event showing the direct effect of ZD6474 on P-gp. Given that ZD6474 is a promising anti-cancer agent that is currently being investigated in clinical trials, the combination of ZD6474 with traditional cytotoxic chemotherapeutics, especially drugs like ADR, docetaxel, and VNR (to which cancer cells readily develop MDR), may be a rational and promising strategy for cancer therapy.

It has been reported that the effects of ZD6474 are synergistic with those of oxaliplatin and docetaxel in cells which do not overexpress P-gp, such as KYSE30 esophageal squamous epithelial cancer cells and HCT-116 and HT29 colon cancer cells (Morelli *et al*, 2005; Troiani *et al*, 2006). A dose-dependent supra-additive effect in cell growth inhibition, *in vitro*, and tumour growth inhibition, *in vivo*, was also observed when ZD6474 was used in combination with paclitaxel or docetaxel (Ciardiello *et al*, 2003). Our study shows that sensitivity to chemotherapeutic agents, in MDR cancer cells, is increased in the presence of clinically relevant concentrations of ZD6474. However, ZD6474 does not modulate chemosensitivity in parental chemosensitive cells. These results seem inconsistent with previous reports (Morelli *et al*, 2005; Troiani *et al*, 2006) and the mechanisms underlying this difference are currently unclear. However, it is noted in these previous reports that the ZD6474 treatment schedule is the key aspect for its combination effect with cytotoxic agents. For example, an antagonistic effect was observed when cells were pretreated with ZD6474 followed by cytotoxic agents. On the contrary, a synergistic effect was evident when cells were pretreated with cytotoxic agents prior to EGFR antagonists (Morelli *et al*, 2005; Troiani *et al*, 2006). In this study, we treated MCF-7 cells with ZD6474 and cytotoxic agents simultaneously. Therefore, it is likely that this treatment schedule lead to the differences observed. Another reason for the apparent discrepancy between our results and previous reports may be that we treated cells in culture for 72 h, whereas Ciardiello *et al* (2003) treated cells in soft agar for 10–14 days. Finally, the different cells used in these studies may also result in different effects of ZD6474 on the cytotoxicity of chemotherapeutics. These data suggest that the treatment schedule must be considered when ZD6474 is used in combination with other cytotoxic agents in the clinic.

Some drug efflux transporters, including P-gp, have an ATP-binding region. The binding of ATP to the transporter's nucleotide-binding site is essential for substrate transport, and the hydrolysis of ATP by P-gp ATPase is critical for restoring the transporter to its active conformational state (Rosenberg *et al*, 2001). Thus, monitoring ATPase activity in cell membrane preparations, or purified membrane proteins, represents a method of identifying those compounds that interact with the drug efflux transporters. P-gp exhibits a highly drug-dependent ATP hydrolysis activity, and a variety of P-gp inhibitors, as well as P-gp substrates, can stimulate ATPase activity (Kerr *et al*, 2001; Garrigues *et al*, 2002; Kitazaki *et al*, 2005). In addition, in the ATPase activity assay, a test molecule is concluded to interact specifically with P-gp if it significantly modulates ATPase activities by >30%, at any one of the concentrations (0.05–50 *μ*M) tested (Garrigues *et al*, 2002). ZD6474 was clearly shown to stimulate ATPase activity, by >30%, in the P-gp-expressing membrane and this stimulation was dose dependent. This suggests that ZD6474 stimulates transporter ATPase activity by directly interacting with P-gp. Although ATPase activity is closely associated with the function of P-gp, an increase in the ATPase activity does not necessarily lead to an enhancement in P-gp function. For instance, VPL is an MDR reversal agent, but it stimulates the ATPase activity of P-gp (Garrigues *et al*, 2002). Another example is the flavonoid compound, 6-prenylchrysin, which is an inhibitor of ABCG2, but stimulates the ATPase activity of ABCG2 (Ahmed-Belkacem *et al*, 2005). Given that the function of P-gp mainly manifests as pumping drugs out of the cells, and ZD6474 was shown to inhibit the efflux of substrates of P-gp, such as ADR, it is believed that ZD6474 reverses MDR by directly inhibiting the function of P-gp.

Tyrosine kinase inhibitors are essentially hydrophobic compounds, which must pass through the cell membrane barrier to reach their intracellular target molecules. It has been reported that tyrosine kinase inhibitors such as STI-571, ZD1839, and EKI 785 interact with ABCG_2_ (Ozvegy-Laczka *et al*, 2004). This modulatory property may make tyrosine kinase inhibitors ideal compounds for use in combination with other anticancer drugs. However, the interaction between ZD6474, an inhibitor of VEGFR, EGFR, and RET tyrosine kinases, and ABC transporters has not been explored. In this study, we show that ZD6474 is capable of reversing P-gp-mediated MDR by directly inhibiting the function of P-gp. More importantly, ZD6474 significantly inhibits the function of P-gp at clinically achievable concentrations (≈2.1 *μ*M) (Holden *et al*, 2005). This implies that simultaneous administration of ZD6474 with cytotoxic agents, especially substrates of P-gp such as ADR or docetaxel, may be of clinical benefit for patients bearing tumours that have P-gp-mediated MDR.

In conclusion, ZD6474 directly interacts with P-gp and inhibits the function of P-gp at clinically relevant concentrations, and this is achieved by stimulation of the P-gp ATPase activity. The ability of ZD6474 to modulate P-gp makes ZD6474 an ideal agent to combine with cytotoxic agents and overcome MDR in the clinic. This strategy is therefore worthy of clinical trial testing for cancer therapy. In addition, ZD6474 blocks several signal transduction pathways that utilise VEGR and EGFR. The precise role of tyrosine kinase receptors in the reversal of P-gp-mediated MDR by ZD6474 warrants further investigation.
